# Secondary Binding Site of CYP17A1 in Enhanced Sampling Simulations

**DOI:** 10.1021/acs.jcim.4c01293

**Published:** 2024-09-26

**Authors:** Tomasz M. Wróbel, Damian Bartuzi, Agnieszka A. Kaczor

**Affiliations:** † Department of Synthesis and Chemical Technology of Pharmaceutical Substances with Computer Modeling Laboratory, Faculty of Pharmacy, Medical University of Lublin, 4A Chodźki St., 20093 Lublin, Poland; ‡ Department of Drug Design and Pharmacology, Faculty of Health and Medical Sciences, University of Copenhagen, Universitetsparken 2, 2100 Copenhagen, Denmark; § Science for Life Laboratory, Department of Cell and Molecular Biology, Uppsala University, 75124 Uppsala, Sweden; ∥ School of Pharmacy, University of Eastern Finland, Yliopistonranta 1, P.O. Box 1627, 70211 Kuopio, Finland

## Abstract

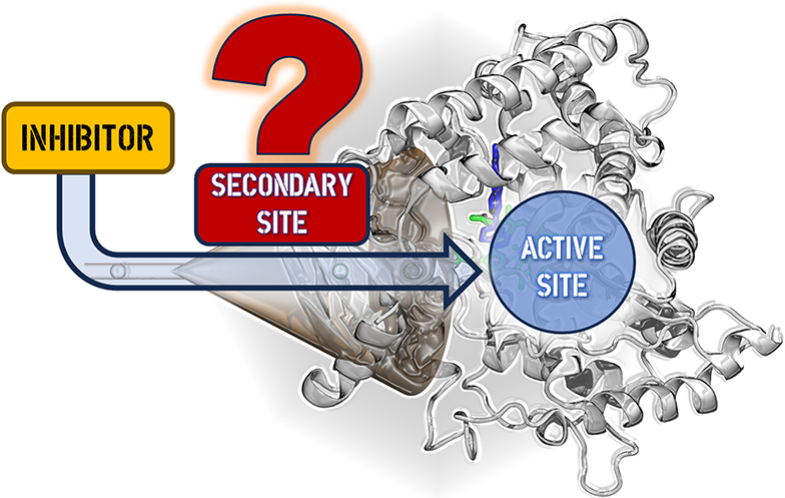

Androgens like testosterone and dihydrotestosterone play a key role in prostate cancer progression, making the enzyme CYP17A1, essential for androgen synthesis, a crucial therapeutic target. Recent studies have revealed electron density at the substrate entry channel, suggesting the presence of a secondary binding site. In this study, we calculated the binding free energy landscape of known ligands at this site using Funnel Metadynamics. Our results characterize this binding site and indicate that nonheme-interacting ligands could effectively bind to CYP17A1, providing a novel approach to the design of CYP17A1 inhibitors.

## Introduction

Cancer remains a significant public health issue and ranks as the second most common cause of death. The increasing prevalence of prostate cancer (PCa) from 2014 to 2019 is particularly worrisome after a downward trend spanning two decades.^1^ While considerable progress has been made in PCa treatment options, the need for more potent therapies remains pressing. Advanced cases of the disease can evolve into castration-resistant prostate cancer (CRPC), which is associated with a grim prognosis.^2^

The androgen receptor (AR) signaling pathway is fundamentally involved in the onset and the progression of PCa.^3^ Androgens, such as testosterone and dihydrotestosterone (DHT), bind to the AR, initiating downstream signaling pathways that promote prostate cancer cell growth and survival. Consequently, the AR signaling pathway has been at the center of therapeutic approaches for PCa.

**Figure 1:**
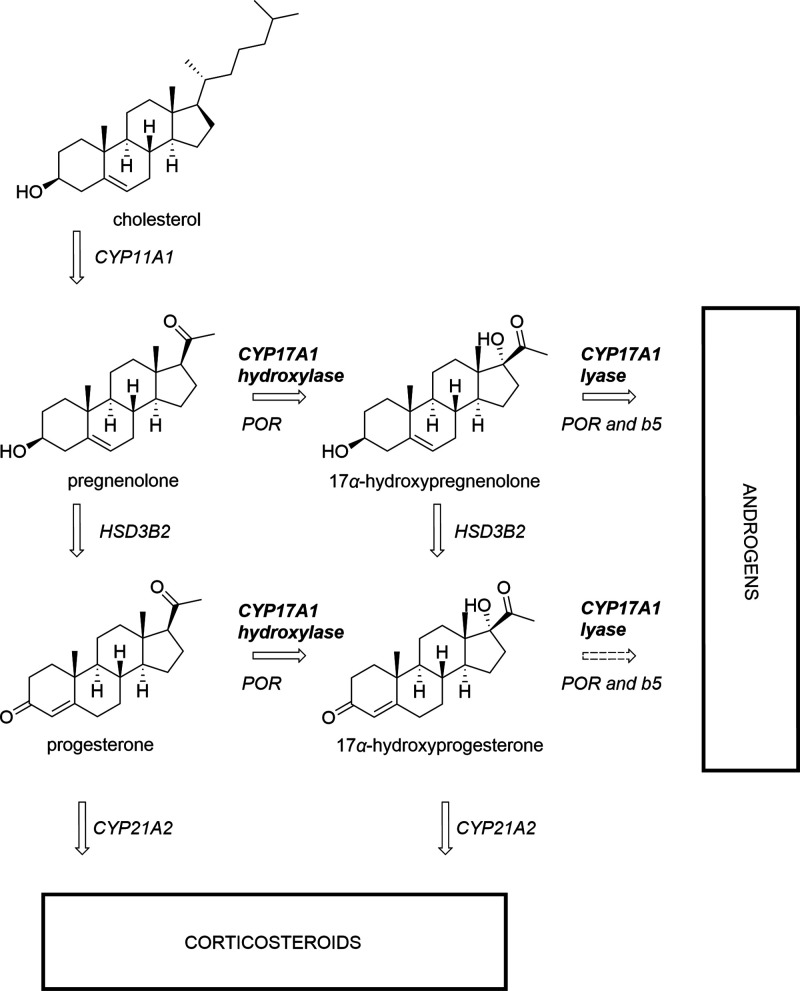
Androgen biosynthesis with emphasis on the CYP17A1 point of action. CYP17A1 functions as a 17α-hydroxylase, converting pregnenolone into 17α-hydroxypregnenolone and progesterone into 17α-hydroxyprogesterone. CYP17A1 can subsequently perform a 17,20-lyase reaction, converting 17α-hydroxypregnenolone into dehydroepiandrosterone, whereas the equivalent reaction with 17α-hydroxyprogesterone is inefficient in humans. POR, Cytochrome P450 reductase; b5, Cytochrome b5; HSD3B2, 3β-hydroxysteroid dehydrogenase type 2.

One potential target in the fight against prostate cancer is CYP17A1, a critical enzyme that facilitates androgen synthesis. This enzyme catalyzes two crucial reactions in androgen production: the 17α-hydroxylase and 17,20-lyase reactions (Figure 1). Inhibiting CYP17A1 results in reduced androgen production, making it an attractive strategy for treating PCa.^4^

Inhibitors of CYP17A1 can be classified into two groups: steroidal, exemplified by abiraterone, and nonsteroidal. To date, abiraterone is the only CYP17A1 inhibitor approved for treating CRPC. However, over time, resistance to abiraterone can develop, necessitating the search for alternative CYP17A1 inhibitors. Nonsteroidal inhibitors, such as seviteronel or orteronel, have demonstrated encouraging results in preliminary studies, but none have yet made their way into clinical use.^5,6^

Both steroidal and nonsteroidal inhibitors commonly interact with the enzyme by coordinating the sp^2^ hybridized nitrogen atom to the heme iron. Indeed, all these compounds contain nitrogen heterocycles, and several solved X-ray ligand-protein complexes clearly indicate this interaction.^7,8^ However, this canonical design has a potential flaw because coordinating the heme iron should, in principle, indiscriminately inhibit both CYP17A1 reactions. Inhibiting the hydroxylase step blocks the production of hydroxylated intermediates, which are also substrates for glucocorticoid biosynthesis.^9^ Insufficient levels of glucocorticoids stimulate the release of adrenocorticotropin hormone, leading to excessive production of mineralocorticoids, which manifests clinically as hypertension, hypokalemia, and peripheral edema.^10^ An ideal CYP17A1 inhibitor should therefore inhibit only the lyase reaction, while also exhibiting selectivity versus other CYP isoforms.^11^

Recent reports suggest that the formation of the iron-ligand complex is not necessary for enzyme inhibition.^12^ Analysis of binding kinetics has shown fast ligand association with the protein, followed by a slower shift into the well-documented nitrogen–iron complex. The ligand presumably occupies a metastable binding site before reaching its destination aligned next to the heme. This finding hints at a possibility of designing molecules that would not interact with the heme iron but still would be able to achieve the desired inhibition, possibly even displaying selectivity unavailable to traditional nitrogen-heterocycle inhibitors. This notion is augmented by earlier X-ray crystallography studies, where electron density corresponding to an unknown ligand was observed in (*S*)-orteronel complexes with CYP17A1.^7^ Similarly, binding of (*S*)-seviteronel displayed comparable results, although the density was weaker in that case. In both cases, X-ray crystallography demonstrated the hallmarks of ligand binding but did not reveal the ligand identity or exact binding mode. Unfortunately, attempts to solve the density in these and subsequent studies failed to provide a high-resolution structure of a ligand bound to the metastable site.^13^

Computational techniques offer insights into structures and events inaccessible by experimental methods. Not only can they provide atomic-resolution models, they can be used to track the structure evolution in time, providing information about mechanisms and energetics of processes. When the time scale of the process becomes prohibitive for regular simulations, enhanced sampling techniques can be used. This is the case for ligand binding simulations. Several techniques for enhanced sampling of the ligand binding process were developed, including collective variable (CV) based methods, such as accelerated weight histogram (AWH),^14^ metadynamics^15^ and its derivatives such as funnel metadynamics (FM),^16^ as well as CV-free methods, such as accelerated molecular dynamics (aMD), Gaussian-accelerated MD (GaMD),^17^ DelPhiForce Steered Molecular Dynamics (DFMD)^18^ and others. These methods along with their applications have been reviewed recently by Shen et al.^19^ Among these, FM provides the possibility of restricting the area available for exploration by ligand, thus decreasing the time of nonproductive sampling.

Intriguing discoveries in the field of CYP17A1 ligands prompted us to explore the possibility of ligand binding at the CYP17A1 secondary binding site. As the above-mentioned studies pinpoint a location of the possible site and indicate a suitable chemical probe, the opportunity of binding mode prediction appeared. Successful identification of crucial interaction would in turn enable prospective drug design efforts, e.g., virtual screening (VS). Therefore, we focused on exhaustive sampling of orteronel in the putative metastable site in order to find its most probable binding mode and pinpoint crucial interactions that could be used in future drug design efforts. Here, we used the FM technique, for the first time for this protein, in enhanced sampling all-atom molecular dynamics simulations to seek plausible explanations of the unclear experimental data and evaluate the druggability of the considered site.

## Materials and Methods

**Figure 2:**
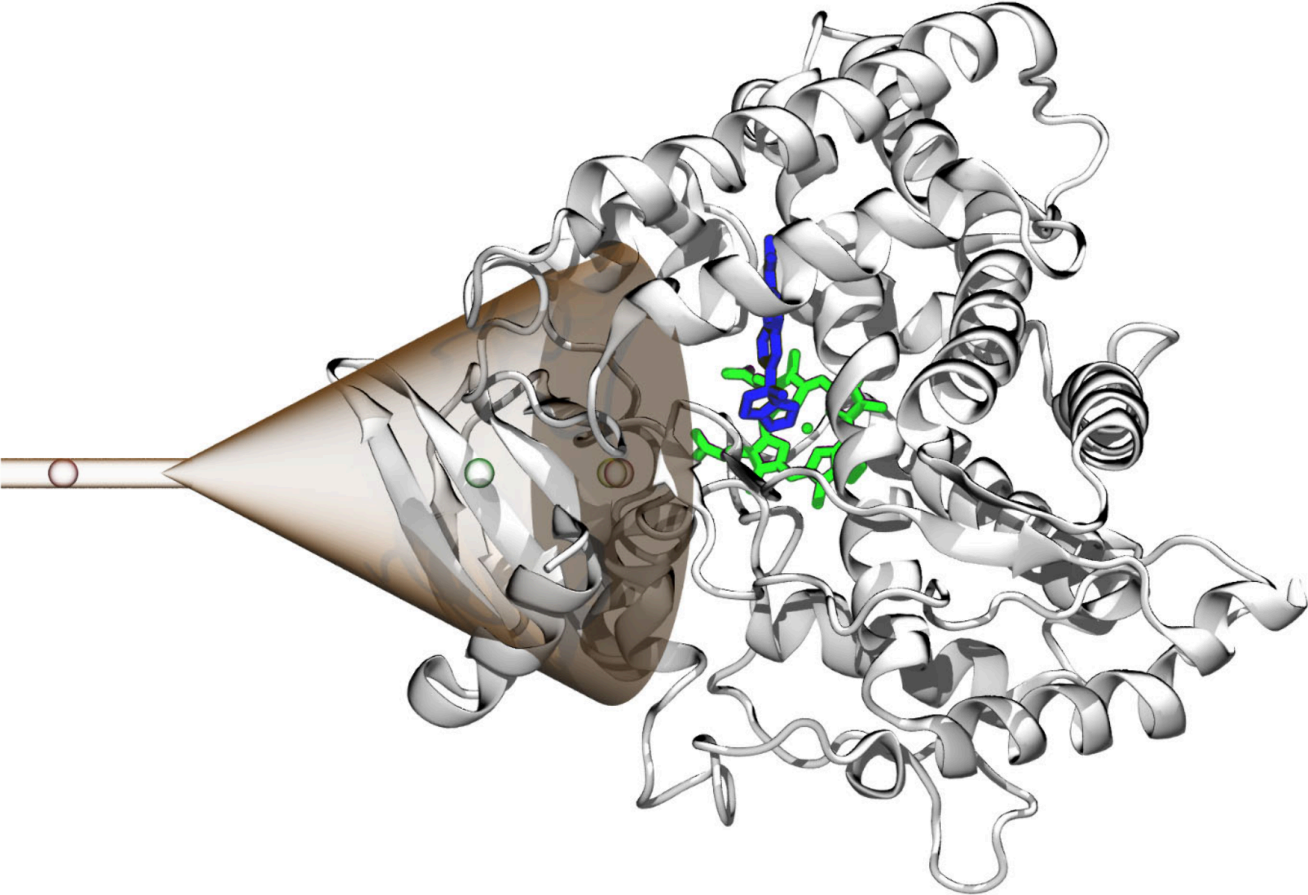
Funnel (red glass surface) defined at the CYP17A1 structure (white cartoon).

To enable exhaustive sampling of protein and ligand flexibility and all possible interaction modes within the area of interest, we decided to use the FM technique.^16^ Gromacs 2022.3 was used as a molecular dynamics’ engine. Amber03 Force Field was used for protein, and General Amber Force Field 2^20,21^ was used for the ligand. Ligand RESP charges were obtained using PyRED,^22^ and ligand topology was generated with ACPYPE.^23^ Heme parameters were prepared based on previous work.^24,25^ To enable enhanced sampling, Plumed 2.8.1 with additional FM module enabled was used.^26^ The simulation setup involved the time step of 2 fs and temperature of 309.75 K. Multiple walker, well-tempered metadynamics approach was used, with five walkers and a Δ*T* value of 4026,75 K. Gaussian height was set at 0.3, width at 0.05 Å and 0.43 rad. Gaussians were deposited every 500 steps. Collective variables were defined as distance along the funnel and torsion between the ligand and I helix (nomenclature according to Pochapsky et al.^27^). The funnel axis was set along the line between the heme and the unknown density (Figure 2). Walls of potential were imposed on RMSD of the protein mainchain, except of the F/G loop, which was completely unrestrained. Free energy values were calculated according to the equations from the original FM paper.^28^ Frames corresponding to the free energy minima on the free energy landscapes were extracted and clustered with a single linkage algorithm. Central frames of the dominating clusters were selected as representative binding modes (Supplementary Files 1 and 2). Molecular visualizations were generated with PyMOL or VMD.^29,30^ Plots were generated with Gnuplot.^31^ While CYP17A1 is a membrane-associated protein, its N-terminal helix anchoring it at the cell membrane is not resolved within the X-ray structure and is connected to the rest of the enzyme by a long random coil. Therefore, the water environment was chosen to avoid possible membrane deformation artifacts and maintain the Gaussian deposition pace of once every 500 steps. This may affect the values of absolute binding free energies but is unlikely to affect the relative differences between protein–ligand interaction energy values. Therefore, the choice of the simulation environment would not interfere with the aim of the study, which is identification of the most favorable binding mode of the ligand.

## Results

**Figure 3:**
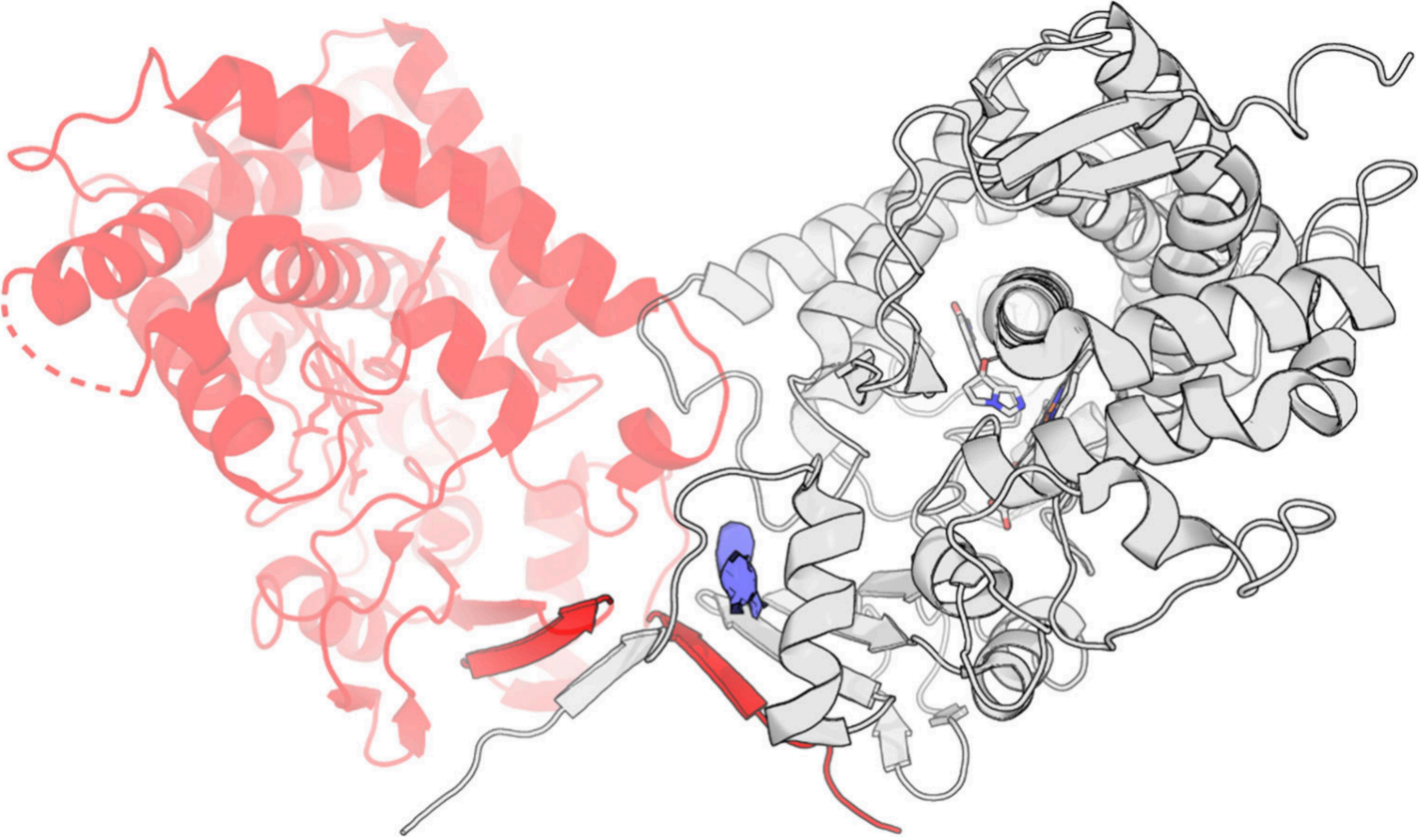
5IRQ pdb record. Red cartoon, (*R*)-orteronel-binding subunit; white cartoon, (*S*)-orteronel-binding subunit; blue surface, unresolved density.

In the paper by Petrunak et al.,^7^ the authors report the presence of a ligand-like density, to which no molecule could be favorably fitted. Visual analysis of the 5IRQ PDB record reveals that the elongated density is located perpendicular to helix A, partially between side chains of N52 and L56 residues, and is surrounded by the N-terminal random coil that turns around it. Notably, this region is located at the interface between crystal subunits, with N-terminal random coils forming β-sheets with the β1 region of another subunit (Figure 3). In full-length wild-type CYP17A1, the random coil serves as a linker to a membrane-embedded helix, anchoring the protein at the membrane surface. It is unlikely that such a dimer is formed natively, and therefore, there was a possibility that the density is only a crystallization artifact. However, its location along the possible ligand entry channel,^32^ combined with the report that CYP17A1 ligand binding involves multiple steps,^12^ suggests the presence of a metastable binding site. Therefore, we prepared an FM setup to sample possible conformations of both orteronel enantiomers and to identify possible binding modes that could be used in further drug design studies. To prevent artifacts and improve the convergence time of the simulations, the N-terminal random coil was removed.

**Figure 4:**
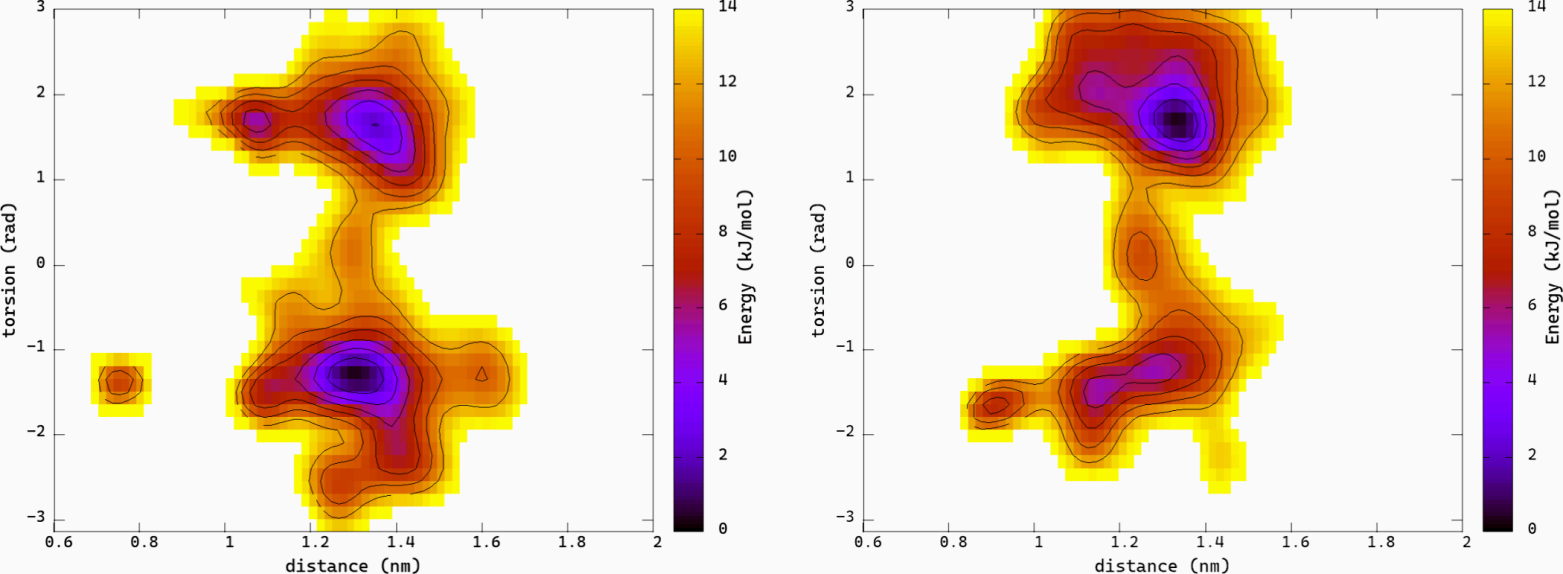
Free energy landscapes calculated for (*S*)-orteronel (left) and (*R*)-orteronel (right).

**Figure 5:**
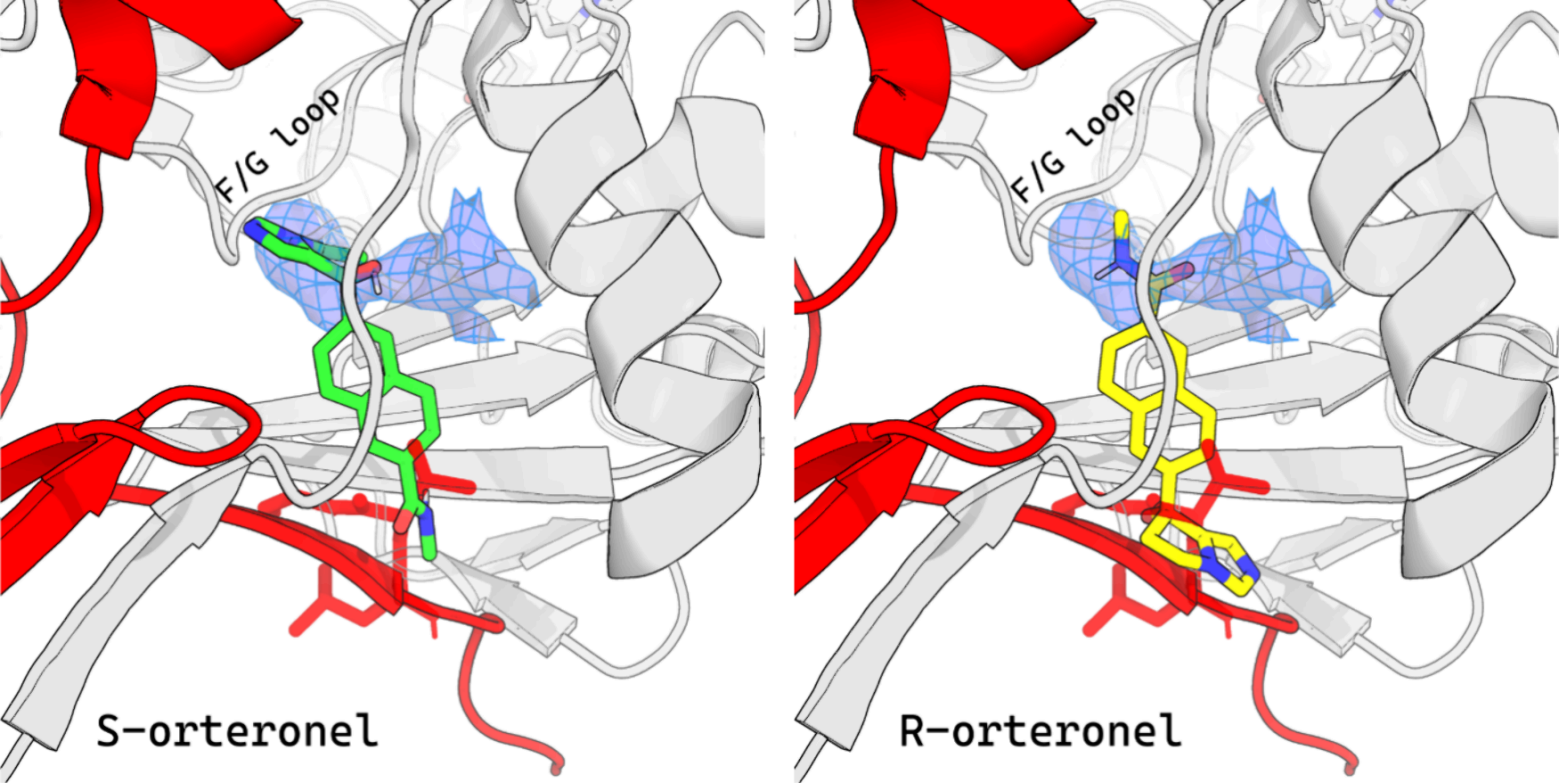
Superimposition of the CYP17A1 structure, with the unresolved electron density reported by Petrunak et al.^7^ as found in the 5IRQ PDB entry, and (*R*)-orteronel (yellow, right panel) or (*S*)-orteronel (green, left panel). The protein unit forming the pocket for the unknown density is shown in light gray, while another unit forming interactions within the crystal lattice is shown in red. Residues overlapping with FM poses of orteronel, which belong to another protein subunit in the crystal lattice, are shown as red sticks.

**Figure 6:**
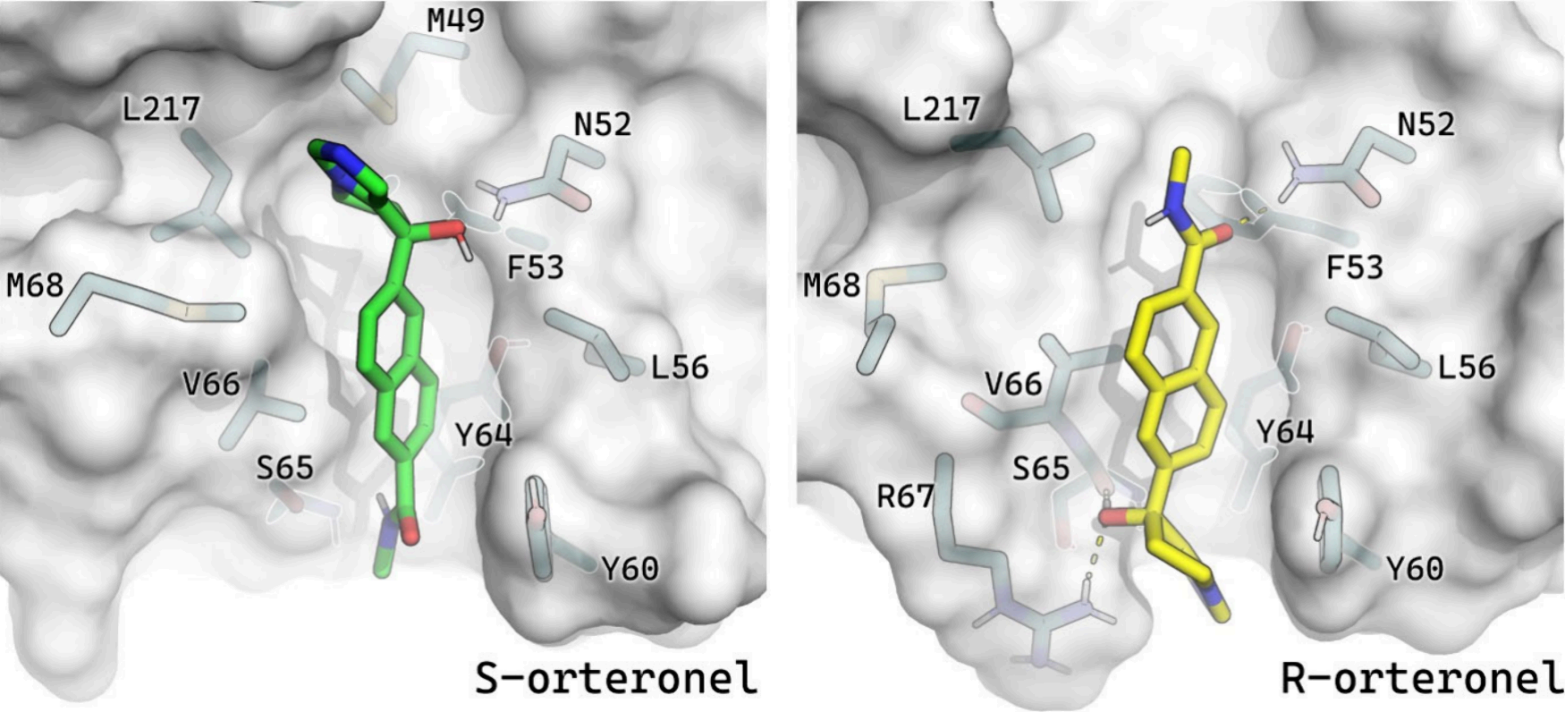
Metastable binding cavity of (*R*)-orteronel (left panel) and (*S*)-orteronel (right panel) identified with FM.

Free energy landscapes obtained for (*S*)- and (*R*)-orteronel are presented in Figure 4. In both cases, two free energy wells can be identified. They correspond to ligand conformation parallel to helix A, and distance values correspond to a cleft between helix A and β1/β3 sheet. Notably, the enantiomer’s propensities to fall into particular wells are different (Figure 4). The ligand poses corresponding to their minima are shown in Figures 5 and 6.

The free energy landscapes of orteronel binding at the metastable pocket show that both isomers can bind in one of two possible orientations, rotated by π radians. However, it is evident that different orientations correspond to the lowest energy states of the different enantiomers: the *S*-enantiomer prefers an orientation at torsion values of approximately −1 rad, while the *R*-enantiomer presents the free energy minimum at 2 rad.

**Figure 7:**
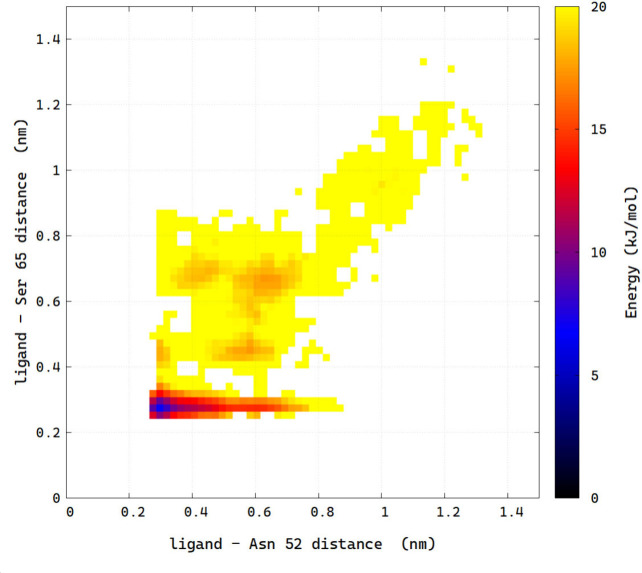
The free energy surface depicting the distribution of distances between the carbonyl oxygen of (*R*)-orteronel and N52 versus the distances between the hydroxyl oxygen of (*R*)-orteronel and the main chain carbonyl oxygen of S65, as observed in the FM simulations.

Superimposition of the X-ray structure and the simulation results clearly show that the most favorable orteronel binding conformations would interfere with the crystal packing (Figure 5). As mentioned, the X-ray structure depicts an oligomer, in which the N-terminal tail of one subunit forms a β-sheet with the β-1 region of another subunit. The ligand molecule in the binding mode predicted with FM would overlap with the N-terminal tail residues of another crystallized protein molecule. Since this coil, in a nontruncated protein, is connected to a transmembrane domain anchoring the enzyme at the cell membrane, such an arrangement is unlikely to occur natively. This overlap explains the difficulties in identifying the corresponding X-ray density, as the ligand was likely to be forced into a suboptimal and less stable binding mode. Notably, an important part of the putative metastable ligand pose and the unresolved X-ray density overlap in the area of a characteristic kink, where (*R*)-orteronel amide group seems to fit better than (*S*)-orteronel heterocyclic rings. Moreover, (*R*)-orteronel engages in several polar interactions, especially with the side chain N52 and the main chain carbonyl oxygen of S65 (Figures 6 and 7). Additionally, a hydrogen bond to R67 could be observed for a fraction of the simulation time.

## Discussion

Prostate cancer poses a significant challenge to public health. Despite the availability of various local and systemic treatments, these often result in substantial side effects and may not guarantee a cure. Hence, there is a demand for novel therapies. The field of CYP17A1 inhibitors has been dominated by ligands containing an sp^2^ hybridized nitrogen atom capable of coordinating to the heme iron and blocking the enzyme active site. To date, efforts in VS have relied on utilizing the known active site. For instance, Bonomo et al. combined a structure-based VS with density functional theory (DFT) to discover potent nonsteroidal CYP17A1 inhibitors.^33^ They used a heme-tailored docking protocol with refinement of the Fe–N interaction by DFT calculations, resulting in the identification of two inhibitors. In another approach, Gumede et al. constructed a 3D-QSAR pharmacophore model based on nonsteroidal CYP17A1 inhibitors and applied it for VS.^34^ Although these efforts yielded several novel chemical entities, their potency was low.

In our current work, we aim to explore the CYP17A1 protein for an alternative binding site. We employ funnel metadynamics, a binding free-energy method that uses a funnel-shaped restraint potential to determine the ligand binding mode and accurately calculate the absolute binding free energy between the ligand and the protein. Funnel Metadynamics is an enhanced sampling method that has proven accurate in binding free energy predictions.^16,28^ Moreover, it was recently applied successfully by Chen et al., who used it to obtain ligand binding modes in the AR. This approach was used in a VS campaign that discovered novel compounds with favorable binding affinities.^35^ Since the quality of protein–ligand complexes is crucial for successful VS, this validates the method’s robustness and usefulness in prospective studies, including the steroid-binding proteins vital in prostate cancer.

The existence of another targetable site would open possibilities for novel structure-based designs, potentially avoiding the lack of selectivity observed in nearly all reported compounds. Recent studies have postulated the existence of a secondary binding site.^13^ It was noted that the F/G loop region contains electron density in some instances of the crystallized ligand-protein complexes. A ligand in that area could possibly stabilize a conformation where the F/G loop is “open”, allowing access to the enzyme active site. This would enable the hydroxylated product to escape before being cleaved by the lyase, as seen in the selectivity between orteronel enantiomers.^7^ Other allosteric effects cannot be ruled out, but our analysis corroborates these previous findings. Unfortunately, FM simulations performed in this study did not reveal the putative “open” state, possibly due to the applied methodology.

The concept of allostery in P450 enzymes, dating back to the 1970s and 1980s, was based on the cooperative behavior of CYP3A4, the most prominent P450 enzyme.^36^ Since then, many other P450 enzymes have been reported to display this cooperativity, making it a common characteristic among P450 enzymes. Other explanations, such as modulation of protein–protein interactions in enzyme oligomers, have also been offered to account for this phenomenon.^37^ However, the concept remained underexplored in hormone-producing P450s.

Research shows that binding to the CYP17A1 protein and other P450s is a multistep process.^12,38^ Moreover, this process appears to be independent of ligand structure, as both nonsteroidal or steroidal compounds (such as abiraterone) have been shown to follow it.^39^ Importantly, CYP17A1 activity is inhibited by initial binding. This process can be envisioned as the ligand first binding to a metastable site before moving to the active site. Metastable binding sites are another concept proposed for exploitation in drug design.^40^ First suggested in the late 1990s for G Protein-Coupled Receptors (GPCRs), this idea was corroborated a decade later by MD simulations showing a pathway traversed by a ligand before binding to the orthosteric site.^41^ It was implied that these temporary binding sites could serve as potential targets for allosteric modulators. However, as these are low affinity binding sites, ligands would need to exhibit increased residence time, possibly achieved through covalent modalities.^42^

An ideal CYP17A1 inhibitor should selectively inhibit the 17,20-lyase reaction while sparing 17α-hydroxylase. This would result in a better pharmacological profile. However, predicting whether an *in silico* designed ligand would exhibit this selectivity is currently difficult, primarily because the nature of hydroxylase/lyase selectivity is not well understood. Conformational changes are believed to play a key role in this selectivity. One such change involves the allosteric effect of cytochrome b5,^43^ while another can result from a ligand influencing the conformation of the F/G loop. Potential conformational changes of the entire protein due to ligand binding to the described site were beyond the scope of our examination. Our study focused on probing the site, providing a framework for further exploitation.

When investigating the binding of putative ligands that do not interact with the heme, it is necessary to consider assays beyond the widely used UV spectroscopy-based methods. Direct coordination of nitrogen-containing heterocycles to the heme iron in the active site of cytochrome P450 enzymes causes a shift of the Soret peak λ_max_ to higher wavelengths. Adding an inhibitor results in characteristic shifts in λ_max_ to 425–435 nm, typical of direct coordination to the heme iron due to a spin shift from the high to low spin state. This behavior is exhibited by type II ligands (inhibitor-like), in contrast to type I ligands (substrate-like), which elicit a shift to high-spin by displacing the axial water ligand without direct coordination. Since no interaction with the heme is expected when operating within the metastable cavity, other methods should be considered, such as surface plasmon resonance (SPR), NMR spectroscopy, isothermal titration calorimetry (ITC), or circular dichroism (CD). However, most published research on CYP17A1 drug discovery often focuses solely on the ligand’s inhibitory effect without examining the binding event. This approach provides essential information about whether the ligand is inhibitory (IC_50_) while deeming the binding mechanism less relevant. Investigating both the binding event and inhibitory effects can provide a comprehensive understanding of the ligand’s interaction with CYP17A1.

## Conclusions

Our research supports previous findings regarding a secondary binding site in CYP17A1. Additionally, we postulate the potential for a structure-based design of CYP17A1 inhibitors targeting this site. This constitutes an innovative approach, proposing a noncanonical binding site for VS in this protein. In summary, we provide a state-of-the-art computational analysis that may aid in the discovery of novel, selective CYP17A1 inhibitors.

## Data and Software Availability

Calculations were performed using the Gromacs software (www.gromacs.org) patched with Plumed (www.plumed.org). Both software packages are available under the GNU Lesser General Public License (LGPL). All simulation parameters were described in the Materials and Methods section, and all relevant molecular structures ((*R*)-orteronel and (*S*)-orteronel in complexes with CYP17A1) are available in the Supporting Information.

## Supporting Information Available

Supplementary File 1: Central frame of the main cluster corresponding to the free energy minimum on the free energy landscape of (*R*)-orteronel binding (PDB)
Supplementary File 2: Central frame of the main cluster corresponding to the free energy minimum on the free energy landscape of (*S*)-orteronel binding (PDB)
Supplementary File 3: Binding free energy convergence plots (PDF)

## Supplementary Material

ci4c01293_si_001.pdb

ci4c01293_si_002.pdb
