# Supplementary material for: Secondary Binding Site of CYP17A1 in Enhanced Sampling Simulations
Source: J Chem Inf Model. 2024 Sep 26;64(19):7679–86. doi: 10.1021/acs.jcim.4c01293 (PMC11480979; doi:10.1021/acs.jcim.4c01293)
Supplement: Supplementary file 3 — ci4c01293_si_003.pdf [file ci4c01293_si_003.pdf]

Supporting information

Secondary binding site of CYP17A1 in enhanced sampling simulations

Tomasz M. Wróbel, Damian Bartuzi, Agnieszka Kaczor

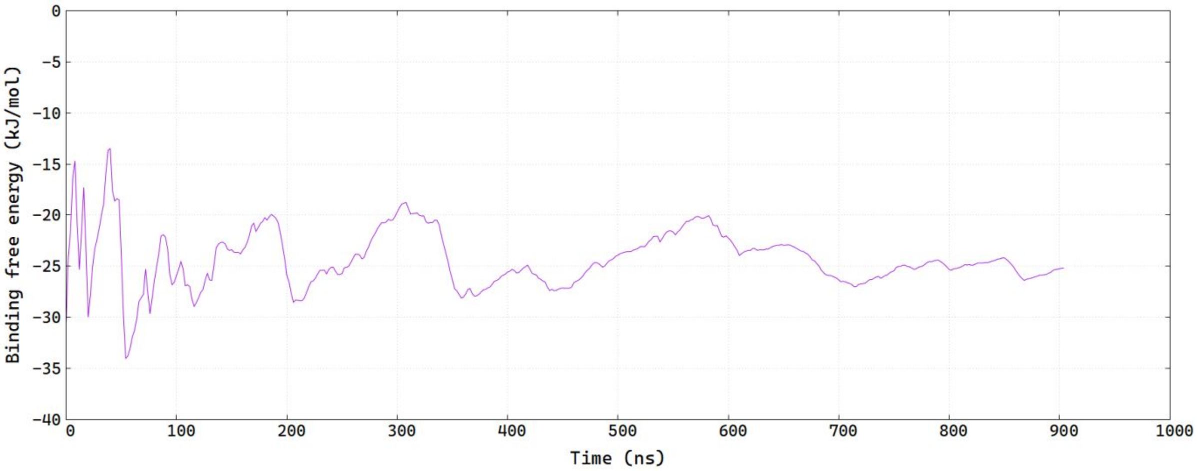

Supplementary figure 1: (R)-orteronel binding free energy convergence over the course of funnel metadynamics simulations

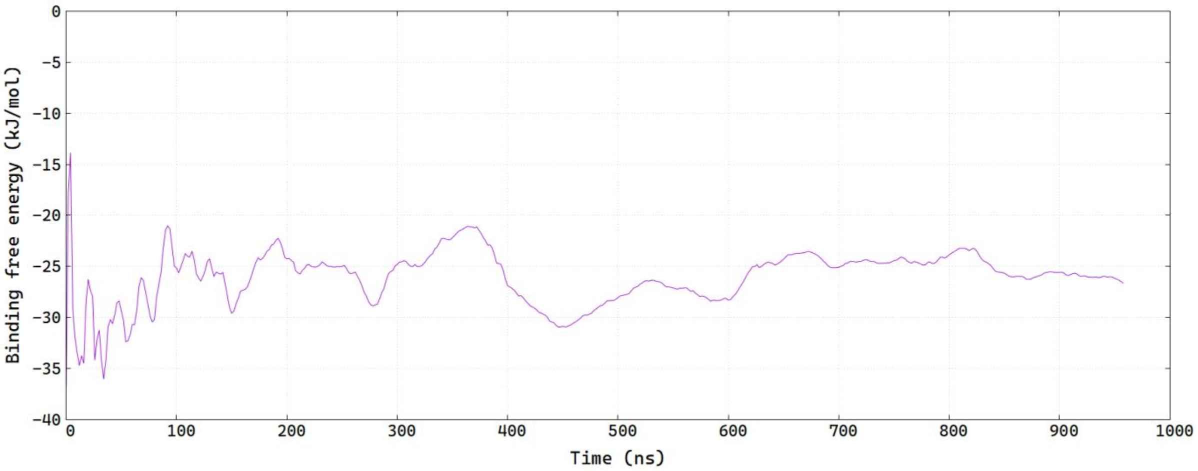

Supplementary figure 2: (S)-orteronel binding free energy convergence over the course of funnel metadynamics simulations
